# Conservation genetics and genetic vulnerability of *Craigia yunnanensis* (Malvaceae), a relict plant species with extremely small populations from Southwest China

**DOI:** 10.1093/aob/mcaf270

**Published:** 2025-10-30

**Authors:** Lei Cai, Yang Liu, Ya-Ling Chen, Zhi-Ling Dao, Wei-Bang Sun, Jing Yang

**Affiliations:** Yunnan Key Laboratory for Integrative Conservation of Plant Species with Extremely Small Populations/State Key Laboratory of Plant Diversity and Specialty Crops, Kunming Institute of Botany, Chinese Academy of Sciences, Kunming, Yunnan 650201, China; Yunnan Key Laboratory for Integrative Conservation of Plant Species with Extremely Small Populations/State Key Laboratory of Plant Diversity and Specialty Crops, Kunming Institute of Botany, Chinese Academy of Sciences, Kunming, Yunnan 650201, China; Kunming College of Life Science, University of Chinese Academy of Sciences, Beijing 100049, China; Yunnan Key Laboratory for Integrative Conservation of Plant Species with Extremely Small Populations/State Key Laboratory of Plant Diversity and Specialty Crops, Kunming Institute of Botany, Chinese Academy of Sciences, Kunming, Yunnan 650201, China; Kunming College of Life Science, University of Chinese Academy of Sciences, Beijing 100049, China; Yunnan Key Laboratory for Integrative Conservation of Plant Species with Extremely Small Populations/State Key Laboratory of Plant Diversity and Specialty Crops, Kunming Institute of Botany, Chinese Academy of Sciences, Kunming, Yunnan 650201, China; Yunnan Key Laboratory for Integrative Conservation of Plant Species with Extremely Small Populations/State Key Laboratory of Plant Diversity and Specialty Crops, Kunming Institute of Botany, Chinese Academy of Sciences, Kunming, Yunnan 650201, China; Yunnan Key Laboratory for Integrative Conservation of Plant Species with Extremely Small Populations/State Key Laboratory of Plant Diversity and Specialty Crops, Kunming Institute of Botany, Chinese Academy of Sciences, Kunming, Yunnan 650201, China

**Keywords:** *Craigia yunnanensis*, endangered, ddRAD-seq, conservation genetics, population demographics, genetic vulnerability

## Abstract

**Background and Aims:**

Conservation genomics research on endangered plants can provide insights for their genetic rescue and adaptive potential. *Craigia yunnanensis* is one of two species of the relict genus *Craigia* that has survived to the present day, and is naturally scattered in southwestern China to northern Vietnam and northern Myanmar. It has been listed as a PSESP species (Plant Species with Extremely Small Populations) in China due to its small populations, scattered distribution and significant human disturbances. Thus, we aimed to use genomic data to conduct research on the conservation genetics and genetic vulnerability of *C. yunnanensis* to provide insights for its population conservation and genetic rescue, and to investigate its potential for adaptation under future climate change.

**Methods:**

We developed genetic markers (SNPs: single nucleotide polymorphisms) for 122 samples from 12 locations based on double digest restriction-site associated DNA sequencing data. Based on commonly used analysis methods in conservation genetics, Stairway Plot 2, genotype–environment association (GEA) and environmental factors, we then made a detailed analysis of its genetic diversity, population structure, genetic vulnerability and demographic history.

**Key Results:**

A total of 1354 common loci with 20 758 SNPs were generated. The results showed that genetic diversity was moderate to high for *C. yunnanensis* when compared to other rare and endangered plant species. Low genetic differentiation is reflected in all populations and can be used to divide them into two genetic clusters. The demographic history revealed that the effective population shows a trough (*Ne* value: 500) during the period 10–20 ka that may correspond to the end of the Last Glacial Period (11.5 ka) and the Last Glacial Maximum (19–26.5 ka), and a period of recovery (*Ne* value: 5500) between 4 and 10 ka. Analysis of genetic vulnerability demonstrates that with non-significant genetic offset (future maladaptabilty with climate change) the ability of the populations to persist into the future will also probably be impacted by other factors such as population size and geographical distance.

**Conclusions:**

Overall, habitat fragmentation and loss of genetic diversity, coupled with severe human interference, have led to the endangered status of *C. yunnanensis*. Additionally, different patterns of genetic vulnerability suggested constructive conservation strategies for different populations. This study also provides additional evidence that the ‘Tanaka–Kaiyong Line’ (TKL) is an obvious phytogeographical boundary in southwestern China.

## INTRODUCTION

Protecting individuals and habitats and elucidating species’ genetic backgrounds are fundamental to the conservation and management of severely threatened species (Moran, 2002; Yang *et al*., 2022*a*; Theissinger *et al*., 2023). Currently, research is lacking on the evolutionary continuity from population to species and population genetics, and difficulties of working on rare organisms mean that genetic rescue and protection of threatened species is very difficult (Frankham *et al*., 2017; Coates *et al*., 2018; Yang *et al*., 2018; Cook *et al*., 2023). In contrast, it is relatively easy to carry out rescue and protection actions such as *in situ*/*ex situ* conservation and returning to nature (Ma *et al*., 2022). The Plant Species with Extremely Small Populations (PSESP) concept recognizes the urgent need to prioritize the rescue and protection of threatened plant groups (Ma *et al*., 2013; Sun *et al*., 2019*a*, *b*, 2021, 2024; Yang *et al*., 2020*a*). Analysis and research into the population genetics, genetic vulnerability and demographic history of PSESPs has been demonstrated to be effective for the conservation and genetic rescue of severely threatened species (Cai *et al*., 2019, 2024; Ma *et al*., 2022; Yang *et al*., 2022*b*; Wang *et al*., 2023; Shen *et al*., 2024; Feng *et al*., 2024*a*  *b*; Liu *et al*., 2024*a*, *b*).

Exploration of the mechanisms underlying the threats facing organisms, analysis of the current adaptability and genetic characteristics of species, and clarification of the conservation needs of species to ensure their sustainability remain key difficulties and the focus of current research into species conservation (Wyse and Kennedy, 2009; Wang *et al*., 2023; Xiong *et al*., 2024). Conservation genetics has transitioned to conservation genomics with the rapid development of molecular technology (Ouborg *et al*., 2010; Ma *et al*., 2022; Cook *et al*., 2023). Meanwhile, research into population genetics and conservation, speciation, population collapse, genetic resilience and adaptation, and demographic and genetic vulnerability has been conducted in several threatened species, including *Cycas* spp., ironwood tree, Chinese hazelnut, *Cinnamomum chago* and *Malania oleifera*. High-quality genomic studies targeting these endangered plant species have revealed the potential of these species for evolutionary adaptation, their genetic diversity and differentiation, their demographic history, and their patterns of genetic vulnerability under future climate change conditions, and also provide insights into their conservation and genetic rescue (Yang *et al*., 2018; Zhang *et al*., 2021; Shen *et al*., 2024; Yang *et al*., 2024; Feng *et al*., 2024*a*, *b* ). Under future climate change scenarios, species that cannot move within their particular niche rely on genetic variation to adapt to new conditions. Genetic vulnerability denotes the potential deficiency in genetic variation necessary to allow the species to adapt to environmental shifts, particularly those brought on by climate change. This deficiency may jeopardize the species’ survival as it encounters these challenges (Fitzpatrick and Keller, 2015; Brauer *et al*., 2023; Zhu *et al*., 2024).

The genus *Craigia* (Malvaceae) is a worldwide relict genus with only two extant species and some fossil species (Wang *et al*., 2021; Li *et al*., 2024). *Craigia kwangsiensis* has unfortunately been assessed as extinct (EX) in the wild in *The China Biodiversity Red List Higher Plants Volume* (2020). The tree *C*. *yunnanensis* (Diantong) is a typical PSESP in Southwest China, with a few individuals found in northern Vietnam and northern Myanmar (Ding *et al*., 2019; Sun *et al*., 2019*a*). *Craigia yunnanensis* has also been subsequently categorized as Endangered (EN) in different checklists (Gao and Sun, 2017; Qin *et al*., 2017). *Craigia yunnanensis* was also listed as a national second-rank protected plant in 1999 and 2021 (www.forestry.gov.cn/main/3457/20210915/143259505655181.html). As a species that requires priority rescue and conservation, the species was also listed as a PSESP at the provincial and national levels in 2010 and 2012 (Sun *et al*., 2019*a*). Fortunately, there have been comprehensive conservation action and scientific studies, including conservation biology, seed and seeding biology, pollination biology and breeding system, genetic diversity and genetic differentiation based on SSRs (simple sequence repeats) and AFLP (amplified fragment length polymorphism), sequencing of the chloroplast genome, *in situ* conservation, *ex situ* conservation and reintroductions (Gao, 2010; Gao *et al*., 2010, 2012; Yang *et al*., 2016, 2020*b*; Wariss *et al*., 2019; Sun *et al*., 2019*a*; Chen, 2020; Chen *et al*., 2020).

In this study, we investigated the population genetics of *C. yunnanensis* using neutral and putatively adaptive SNPs (single nucleotide polymorphisms), and analysed the genetic diversity, population structure, genetic vulnerability and demographic history of this species. We have identified factors that may pose threats to the species at the genomic data level, and therefore propose detailed and targeted conservation strategies for *C. yunnanensis*. Our investigation into *C. yunnanensis* provides a case study for the conservation of similar endangered relict species, and also offers a new perspective for understanding the formation and maintenance mechanisms of such PSESPs.

## MATERIALS AND METHODS

### Field sampling and DNA extraction

**Fig. 1.**
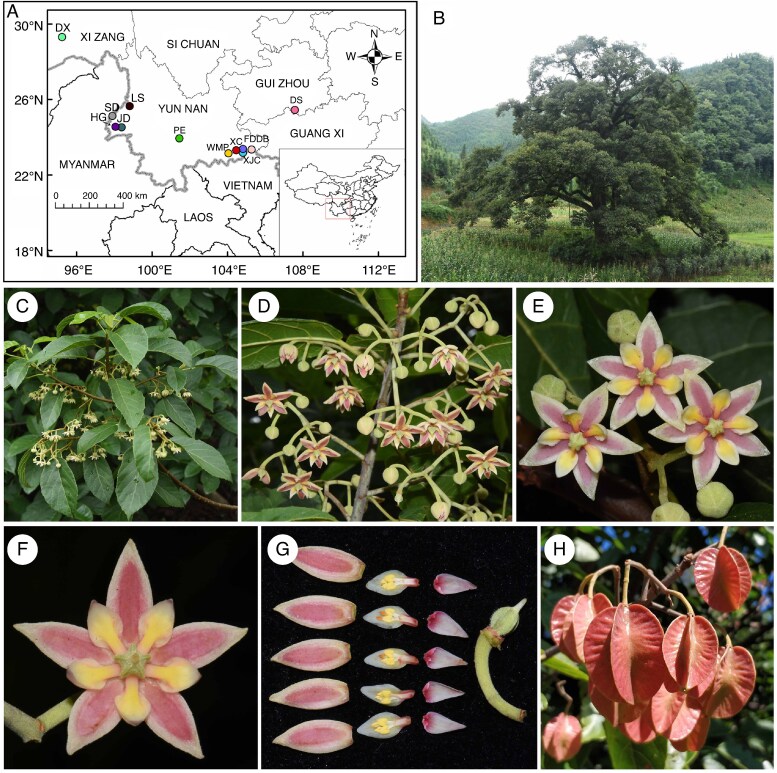
Geographical distribution of 12 sampled populations and morphological characteristics of *Craigia yunnanensis*. (A) Geographical distribution of 12 sampled populations. (B) Wild plant in farmland. (C) Branches with flowers. (D–F) Flowers. (G) A dissected flower. (H) Fruits.

All of the 122 *C. yunnanensis* leaf samples were collected from the wild from localities across Guizhou, Yunnan and Xizang provinces, during 2017–2021. The collections cover all 12 known Chinese populations from east to west and the population designations reflect this process (Fig. 1; Table 1). The sampling strategy was as follows: if the number of individuals in an independent population was ≤10, all were collected; if the number of individuals in the same population was >10, collection was conducted at intervals of more than 30 m. Healthy, clean leaf tissue was collected from trees in their natural habitat for DNA studies; leaves were quickly packed into silica gel desiccant and were stored at −20 °C until sequencing. Voucher specimens were collected and are stored in the herbarium KUN. The extraction of genomic DNA from the silica gel-dried leaf material was carried out using a CTAB method (Doyle and Doyle, 1990; Liu *et al*., 2020). We then sequenced all samples of *C. yunnanensis* using a double digest restriction-site associated DNA sequencing (ddRAD-seq) technique. JieRui BioScience Co. Ltd (Guangzhou) conducted the DNA extraction and ddRAD-seq library preparation.

**Table 1.** Sampling information of 12 *Craigia yunnanensis* populations.

Population code	Population location	Latitude (N)	Longitude (E)	Altitude (m)	No. of samples	Total no. of individuals
DS	Dushan, Guizhou	25°28′	107°37′	850	17	40
DB	Dongbao, Malipo, Yunnan	23°20′	105°16′	1385	4	10
XJC	Xijinchang, Malipo, Yunnan	23°02′	104°46′	1444	19	35
FD	Fadou, Xichou, Yunnan	23°22′	104°46′	1562	16	16
XC	Xichou, Yunnan	23°22′	104°35′	1395	4	4
WMP	Wenshan, Maguan, Yunnan	23°08′	104°03′	1668	9	9
PE	Heping, Mojiang, Pu’er, Yunnan	23°27′	101°22′	1859	2	2
JD	Jiangdong, Mangshi, Yunnan	24°31′	98°23′	1865	10	11
HG	Huguo, Longchuan, Yunan	24°33′	98°04′	1652	5	5
SD	Sudian, Yingjiang, Yunnan	24°36′	97°36′	1327	12	14
LS	Gaoligong, Lushui, Yunnan	25°40′	98°49′	1715	20	91
DX	Dexing, Motuo, Xizang	29°19′	95°10′	998	4	7
Total					122	244

### Sequencing and SNP calling

The ddRAD-seq database construction process was then initiated. Each qualified DNA sample (200 ng 10 µL) was mixed thoroughly with 10 µL of mixed double digestion restriction enzymes (EcoRI + MseI) using a 20-µL pipette tip. The samples were then incubated for 5 h at 37 °C, then for 20 min at 65 °C, and finally incubated indefinitely at 12 °C. Agarose gel electrophoresis was then used to separate 5-µL samples of each enzyme digestion product. Qualifying digested fragments were sequentially ligated to EcoRI with specific barcode labels and MseI adapters using T4 DNA ligase (NEB). Samples were mixed well using a 200-µL pipette tip, then incubated for 8 h at 37 °C, for a further 20 min at 65 °C, and finally incubated indefinitely at 12 °C. The sample digestion/connection products with different barcodes were mixed after the connection, and subjected to agarose gel electrophoresis (Omega). Fragments with a target length of 350–550 bp were identified and excised from the gel. Each library was PCR-amplified to meet the requirements of sequencing concentration, and was further sequenced at 0.5 G per sample using PE 150 mode on an Illumina Novaseq platform.

Analysis of the ddRAD seq data, including quality control and assembly of loci, was conducted by the software Stacks v.2.4 (Rochette *et al*., 2019). The module process_radtags was utilized to conduct quality checks on the offline data, employing parameters such as len_limit set to 140 bp and retain read length to 135 using the parameter -t 135. Based on statistical analysis of the sample data, the single sample R1 data were clustered and deduplicated using the ustacks module to form a locus. The cstacks module was used to generate catalogue files for loci obtained from all individuals, and a total of 1 162 568 catalogue loci were obtained. The sstacks module was used to create the matches file by comparing the SNPs, alleles, and tag data of an individual sample against the catalogue file. The tsv2bam and gstacks modules were then run to prepare for format conversion before SNP calling. The populations module with certain parameters (|−*r*| = 0.5, |−*p*| = 8) was used to call the common SNP loci. We filtered out sites with low frequencies, and recommend a min_maf value of 5–10 %, but we need to choose the default value and keep it if available. The main process of SNP filtering includes: first removing sequencing/alignment errors, then removing individuals/loci with excessive deletions, then removing depth anomalies, allele balance anomalies, repeat regions, sites showing Hardy–Weinberg deviation, and finally retaining only biallelic SNPs. After obtaining the corresponding SNP loci, they can be used to analyse various genetically related studies.

### Genetic diversity, genetic structure and factors driving gene flow

Tajima’s *D* statistic was determined utilizing the vcftools software v.0.1.16, applying a 95 % confidence interval ranging from −1.795 to 2.052, to assess neutrality across all loci and to identify sites under selection (Tajima, 1989; Danecek *et al*., 2011). Subsequently, we employed BayeScan v.2.1 to deduce the loci undergoing selection within each of the 12 populations that were sampled (Foll and Gaggiotti, 2008). Population genetic statistics, including nucleotide diversity (π), expected heterozygosity (*H*_e_), observed heterozygosity (*H*_o_), fixation index (*F*_ST_) and inbreeding coefficient (*F*_IS_), were calculated at each sampling location and in each genetic group using the ‘populations’ module in the Stacks pipeline (Catchen *et al*., 2017).

Admixture in conjunction with Bayesian-based analysis was used to infer the population structure of *C. yunnanensis* (Alexander *et al*., 2009). To determine the optimal value of *K*, we tested numbers of clusters from 1 to 20 and selected the optimal value based on the lowest cross-validation (CV) error rate. Principal component analysis (PCA), based on the poppr software package, and discriminant analysis of principal components (DAPC) were both performed to validate the analysis of population structure (Jombart *et al*., 2010; Kamvar *et al*., 2014).

The vegan package v.2.6.2 and the geosphere package v.1.5.18 were used for analysis of the relationships between populations with geography, environment and genetics as factors. An isolation-by-distance (IBD) test was conducted to assess how geographical distance affects the genetic connectivity among individuals, and an isolation-by-environment (IBE) analysis was conducted to evaluate the impact of environmental distance on genetic diversity among individuals (Hijmans *et al*., 2021). At the same time, environmental parameters were derived from the current climatic data sourced through WorldClim v.2.1. Genetic variation among populations was quantified utilizing all available SNP loci. The degree of genetic differentiation, denoted by *F*_ST_, was determined using the *F*_ST_/(1 − *F*_ST_/) formula, meaning that data were readily interpretable and directly comparable. The statistical robustness of our correlation analysis was assessed by the computation of Spearman’s rank correlation coefficient, complemented by 9999 permutations.

### Inference of demographic history

In the absence of a reference genome, Stairway plot 2 is generally considered to be a highly accurate non-parametric approach for inferring population history, compared to other methods such as the MSMC or PSMC (Liu and Fu, 2015, 2020; Wu *et al*., 2024). We used Stairway plot 2 to deduce the population history of *C. yunnanensis*, assuming a mutation rate of 1.2e-8 per locus per generation (based on unpublished data for *C. yunnanensis*) and a generation time of 8 years, as inferred from cultivation records in Kunming and an *ex situ* conservation base in Maguan County, Yunnan Province (Liu and Fu, 2020). The ANGSD v.0.936 software was utilized to construct the site frequency spectrum (SFS) for *C. yunnanensis* by employing the functions doSaf and realSFS (Korneliussen *et al*., 2014; Yang *et al*., 2022*a*). Site frequency spectrawere created for the entire set of samples as well as for each genetic group, derived from the analyses of population structure. The effective population size was then visually represented through time using Stairway Plot 2. For the accuracy of the model prediction, we removed all sites with a minor allele frequency (MAF) that was too low (or too high).

### Screening for environmental factors and outlier loci

Data from 19 environmental factors (1970–2000) were obtained from WorldClim v.2.1, with a resolution of 2.5′ (Fick and Hijmans, 2017). To ensure the robustness of subsequent regression analyses, only populations with a sample size of ≥4 individuals were included for further analysis; if there are too few samples, analysis of the model will be inaccurate. Based on the environmental factor data, the R packages sp v.1.4.7 (Bivand *et al*., 2013) and raster v.3.5.11 (Robert *et al*., 2023) were used to extract BIO1–19 for 11 populations. By calculating the correlation between each environmental factor and using MaxEnt v.3.4.3 to analyse the weight of each factor (Phillips and Dudik, 2008) (Supplementary Data Fig. S1), and combining the results of both, environmental factors with a correlation coefficient |*r*| < 0.75 (Fig. S2) and a significant contribution to the species distribution were identified. Among the environmental factors considered, BIO4 accounts for temperature seasonality, BIO6 reflects the minimum temperature of the coldest month, BIO7 signifies the annual temperature range, BIO18 pertains to precipitation during the warmest quarter and BIO19 refers to rainfall in the coldest quarter.

Genotype–environment association (GEA) approaches allow for associations between genomic data and environmental factors. In this paper, two approaches, RDA (redundancy analysis) (Rellstab *et al*., 2015) and LFMM (latent factor mixed modelling) (Caye *et al*., 2019), were used to analyse associations between SNPs and five environmental factors to screen for outlier loci. RDA was based on the vegan v.2.6.2 R package (Dixon, 2003), and input files were obtained by formatting the raw vcf files using plink v.1.9 to obtain csv files suitable for statistical analysis (Purcell *et al*., 2007). The input files were not allowed to have missing values, which were replaced with the non-missing value that occurred most frequently in the column (Forester *et al*., 2018). A randomized 999th ANOVA was then performed using the RDA model to test whether environmental variables were significant in explaining species distributions. To select environmentally relevant SNPs, the loci identified at the threshold of ±3 deviations.d. (with a two-tailed *P*-value of 0.0027) were considered as candidates (Liu *et al*., 2024*a*, *b*). LFMM analyses were based on the R package LEA v.3.2.0 (Frichot and François, 2015), with the input file in VCF format, and using the vcf2lfmm function to convert the VCF file into a format that LFMM could handle. The impute function was used to fill in any missing genetic data in the file. To control the FDR (false discovery rate), the *P*-value of each SNP locus was corrected, and finally the SNP corresponding to the value with a *P*-value of <0.001 was selected as the locus where the environment was most closely associated with the gene (Wang *et al*., 2023).

### Genetic vulnerability under climate change

To assess the genetic vulnerability of endangered species to climate change, the gradient forest (GF) model within the R package GRADIENTFOREST was used for predictions (Ellis *et al*., 2012). We consolidated the GEA loci identified through the methods mentioned above to create a comprehensive dataset of outliers. Subsequently, we randomly selected 1000 loci from across all SNPs to serve as a reference dataset. To minimize false-positives, we removed any loci from both datasets where the MAF was below 0.1. Based on these two datasets, we next calculated the allele frequencies across different populations for each locus, which were then used as input files for model construction. The model involved performing 2000 regression tree tests for each SNP locus. To provide a comparative analysis of the future genetic offset relative to the current state, we evenly selected 62 830 loci from within the study area for environmental factor data extraction.

We downloaded two emission scenarios (SSP126 and SSP585) under the climate model CNRM-ESM 2-1 from WorldClim v.2.1 for the period 2081–2100 (Seferian *et al*., 2019). From these, we extracted five environmental factor datasets to predict the environmental changes and to assess how these changes would affect the environmental adaptation alleles compared to the current climate scenario. Ultimately, we assessed the genetic vulnerability of the two datasets across different climate scenarios. The resulting data were then imported into ArcGis v.10.8 (Environmental Systems Research Institute, Redlands, CA, USA; http://www.esri.com) and analysed using the inverse distance weighted (IDW) method to interpolate and visualize the genetic vulnerability.

## RESULTS

### Processing of ddRAD-seq data

Following the removal of poor-quality reads and adapter sequences, a total of 220 349 109 clean reads were left for analysis, averaging 1 806 140 reads for each sample. In total, 4 842 160 loci were obtained after trimming and clustering. The average coverage per locus for all samples was 27.12×, with values ranging from 8.15 to 109.23 (Supplementary Data Table S1). We obtained 1 162 568 catalogue loci after cstacks module processing of all 122 samples of *C. yunnanensis*. A total of 1354 common loci with 20 758 SNPs (201 loci under selection with 5274 selected SNPs, 1153 neutral loci with 15 484 SNPs) were identified using Tajima’s *D*. Selection and neutral loci were also evaluated using Bayescan v.2.1. We were unable to distinguish all population structures from analysis of the 201 selected loci (including 5274 SNPs), and thus we selected all common loci (including 20 758 SNPs and neutral loci with 15 484 SNPs) for further analysis.

### Genetic diversity, genetic structure and factors driving gene flow

The genetic diversity parameters of *C. yunnanensis* at the population level are summarized in Table 2. In the ‘all loci’ dataset, the count of private alleles varied from 42 in population DS to 941 in DX, with a mean of 325.67, while the genetic diversity (π) values ranged from 0.0347 in PE to 0.0812 in DB, averaging 0.0528 across all populations. Observed heterozygosity (*H*_o_) varied between 0.0212 (SD) and 0.0364 (FD), averaging 0.0295. Meanwhile, expected heterozygosity (*H*_e_) within populations fluctuated from 0.0205 (PE) to 0.638 (DB), with an average of 0.0460. The inbreeding coefficient (*F*_IS_) ranged from 0.0119 (PE) to 0.1297 (XJC), with an average of 0.0708.

**Table 2.** Population genetic diversity indices of *C. yunnanensis* (π, genetic diversity; *H*_o_, observed heterozygosity; *H*_e_, expected heterozygosity; *F*_IS,_ inbreeding coefficient).

Population ID	Private alleles	Mean no. of individuals per locus	π	*H*_o_	*H*_e_	*F*_IS_
DS	42	12.2348	0.0465	0.0228	0.0446	0.0941
DB	187	2.5483	0.0812	0.0289	0.0638	0.0873
XJC	794	13.8465	0.0587	0.0326	0.0564	0.1297
FD	183	10.0966	0.0545	0.0364	0.0516	0.0867
XC	156	2.5488	0.0683	0.0303	0.0536	0.0630
WMP	335	6.3357	0.0575	0.0316	0.0526	0.0785
PE	77	1.2627	0.0347	0.0267	0.0205	0.0119
JD	294	8.1045	0.0432	0.0333	0.0401	0.0365
HG	126	3.8576	0.0460	0.0295	0.0398	0.0352
SD	245	7.4596	0.0554	0.0212	0.0514	0.1208
LS	528	15.8092	0.0417	0.0280	0.0401	0.0802
DX	941	3.2845	0.046	0.0323	0.0376	0.0256
Mean	325.67	7.2824	0.0528	0.0295	0.0460	0.0708

**Fig. 2.**
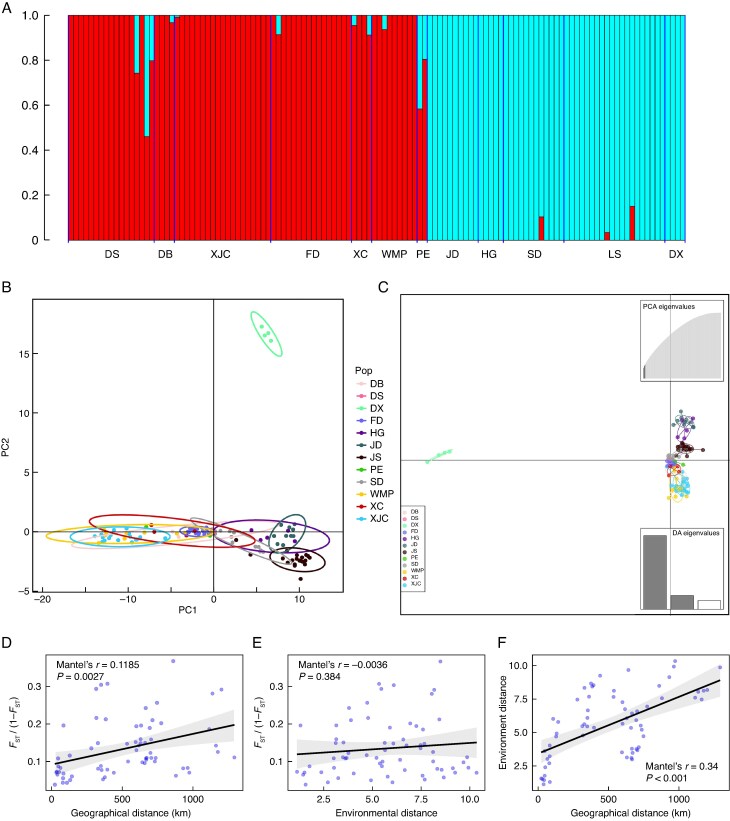
(A) Population structure of *Craigia yunnanensis* from 12 subpopulations inferred by Stacks data sets. Admixture results with *K* = 2. (B) Principal components analysis scatterplots based on the PCA. (C) Principal components analysis scatterplots based on the DAPC. (D) Mantel test between genetic distance and geographical distance. (E) Mantel test between genetic distance and environmental distance. (F) Mantel test between geographical distance and environmental distance.

The population genetic structure of *C. yunnanensis* was analysed using the Admixture method. Upon setting the *K* value range from 1 to 20, the optimal *K* value was identified as 2 (Fig. 2A), corresponding to the CV error value of 0.36368 (Supplementary Data Fig. S3, Table S2). To address the possibility of bias in the selection of *K*, we further examined the genetic structure of *C. yunnanensis* for *K* values ranging from 3 to 7, but under these scenarios, both the populations and individuals were found to be chaotic and could not be formed into related independent clusters (Fig. S4). The genetic structure of the populations was therefore divided into two distinct clusters supported by data from all loci and neutral loci, although these two clusters were difficult to separate using data from selected loci. Both the PCA and DAPC clustering analyses indicated that the 12 study populations should be divided into two clusters (Fig. 2B, C). It is clear that these two clusters are almost bounded by the PE population distributed in the Ailao Mountain area.

Genetic differentiation was mainly reflected through the pairwise *F*_ST_ values, which are given in Table 3. Among the populations, DX and PE exhibited the maximum *F*_ST_ value of 0.3680, while FD and XJC displayed the lowest F_ST_ value, 0.0384. Our analysis showed a weak relationship between geographical distance and genetic variance (Mantel’s *r* = 0.1185, *P* = 0.0027) (Fig. 2D), underscoring the impact of geographical separation on genetic divergence. Interestingly, the correlation between environmental factors and genetic variance did not reach significance (Mantel’s *r* = −0.0036, *P* = 0.384) (Fig. 2E). Moreover, a significant association was detected between environmental factors and geographical distance, with a Mantel’s correlation coefficient (*r*) of 0.34 and a *P*-value of <0.001 (Fig. 2F), highlighting the role of geography in shaping environmental conditions, which in turn can influence genetic patterns.

**Table 3.** Genetic differentiation coefficient (*F*_ST_) among each pair of sampling sites.

Sampling location	DB	XJC	FD	XC	WMP	PE	JD	HG	SD	LS	DX
DS	0.0803	0.0573	0.0515	0.0776	0.0697	0.0975	0.0815	0.0826	0.0698	0.0623	0.1109
DB		0.0717	0.0864	0.01964	0.1164	0.3075	0.2102	0.2570	0.2017	0.1586	0.2916
XJC			0.0384	0.0646	0.0438	0.0769	0.1.030	0.1092	0.1089	0.1042	0.1248
FD				0.0724	0.0552	0.0876	0.1081	0.1220	0.0993	0.0950	0.1423
XC					0.1080	0.2939	0.1958	0.2430	0.1898	0.1478	0.2809
WMP						0.1345	0.1511	0.1592	0.1487	0.1298	0.1851
PE							0.2163	0.3045	0.2126	0.1492	0.3680
JD								0.0708	0.0679	0.0498	0.1421
HG									0.0823	0.0593	0.1941
SD										0.0436	0.1470
LS											0.0998

### Demographic history

**Fig. 3.**
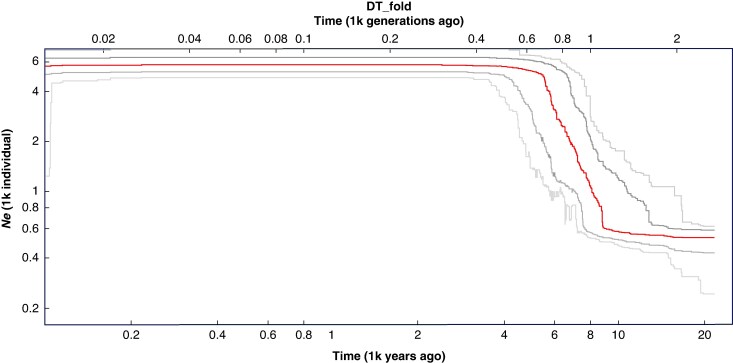
Demographic history of *Craigia yunnanensis* inferred by Stairway Plot 2.

From the demographic history of *C. yunnanensis* inferred using Stairway Plot 2, it was difficult to accurately determine any bottleneck events in this species, though some bottleneck events should occurred before 20 ka. However, we were able to infer that the effective population size (*Ne*) was very low, with a value of about 500, between 10 and 20 ka. Subsequently, the population of *C. yunnanensis* is predicted to have experienced a period of recovery between 4 and 10 ka. Since then, the effective population size of *C. yunnanensis* has remained stable at an estimated value of 5500. Despite the absence of a reference genome, differences in effective population size were observed, and similar demographic patterns were inferred using a generation time of 8 years (Fig. 3).

### Screened environmental factors and outlier loci

Our comprehensive analysis yielded five environmental factors that could be used for subsequent analyses, BIO4, BIO6, BIO7, BIO18 and BIO19, of which two were related to precipitation and three to temperature. Based on the two GEA methods, a total of 2904 outliers were screened. Of these, 1182 outlier loci were identified using RDA, of which 304 corresponded to BIO4, 543 corresponded to BIO6, 12 corresponded to BIO7, 166 corresponded to BIO18 and 157 corresponded to BIO19 (Supplementary Data Table S3). A total of 1739 loci were identified by LFMM as being associated with environmental factors, including BIO4 (262), BIO6 (200), BIO7 (231), BIO18 (687) and BIO19 (1443) (Table S4). Of these, 17 loci were common to both methods tested. To ensure the reliability of the screening results for non-independence of environmental variables and outlier loci, we tested the selected environmental variables. After preliminary screening, we set a threshold (such as |*r*| < 0.75) to remove highly correlated variables, and then used LFMM for environmental correlation analysis.

### Genetic offset

**Fig. 4.**
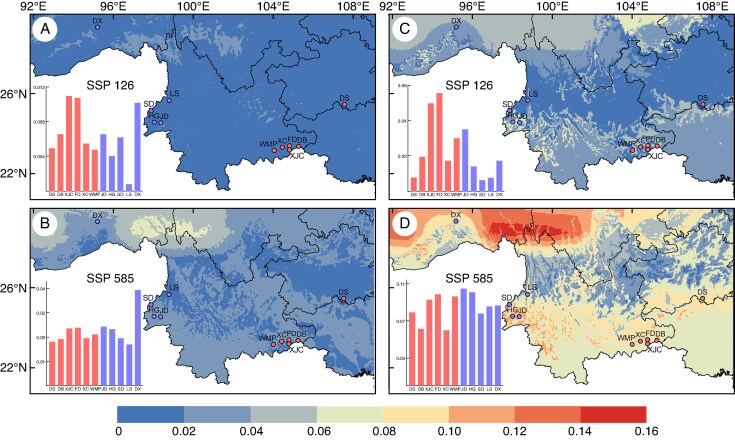
Predicted genetic offset of *Craigia yunnanensis* in the year 2100 under the SSP126 and SSP585 scenarios based on all SNPs (A, B) and adaptive SNPs (C, D). Higher values indicate more severe genetic vulnerability; the bar chart corresponds to the genetic offset of different populations in different scenarios.

Genetic offset quantifies the degree of ‘mismatch’ between existing genome composition and future environmental demands (Fitzpatrick and Keller, 2015). Genetic vulnerability was predicted for all loci as well as for outlier loci under two climate emission scenarios for 2081–2100. Based on the offset values of different populations obtained in this study, the offset values between the western lineage and the eastern lineage are not significantly different. Different vulnerabilities are indicated by the values, with larger values indicating a stronger genetic offset, which means that the population has poor adaptability to future climate and high risks (Fig. 4; Supplementary Data Fig. S5). Examining the graphical representation, we found that population DX has a higher genetic offset relative to other populations based on all SNPs, and faces a greater threat from environmental change in the future, which could result in a reduction of the population size or potentially lead to extinction in the face of climate change. The response of outlier loci to climate change is not significant.

## DISCUSSION

By analysing the conservation genetics and genetic vulnerability of *C. yunnanensis* with genomic data, we sought to inform its population conservation and genetic rescue and to explore its adaptive potential under future climate change. Analyses of *C. yunnanensis* were based on 1354 common loci containing 20 758 SNPs. Compared with other rare and endangered plants, the species exhibited moderate to high genetic diversity, while all populations displayed low genetic differentiation and could be assigned to two genetic clusters owing primarily to geographical boundaries. Demographic history reconstructions indicate that the effective population size reached a trough at 10–20 ka, coinciding with the end of the Last Glacial Maximum (19–26.5 ka) and the end of the Last Glacial Period at ∼11.5 ka. Under projected future climates, the genetic offset among groups was found to be negligible, pointing to limited genetic vulnerability with future anticipated climate change.

### Genetic diversity

Genetic diversity serves as the ultimate manifestation of biodiversity, since it constitutes the foundational basis of biological variation within species (Theissinger *et al*., 2023). Understanding genetic diversity at the population level, especially for threatened species, is particularly important in the field of conservation biology (Xiong *et al*., 2024). Scattered distribution, fragmented habitats, efficiency of pollinating insects and lack of natural regeneration can all affect the stability of genetic diversity of endangered species (Gao *et al*., 2012; Cai *et al*., 2024). The genetic diversity value of *C. yunnanensis* (0.0347–0.0812) was not significantly different from that of *Magnolia fistulosa* (0.030–0.068) (Yang *et al*., 2022*a*) and *Rhododendron cyanocarpum* (0.0634–0.0683) (Liu *et al*., 2020), but lower than that of *Cycas chenii* (0.113–0.145) (Wu *et al*., 2024) and *Corybas taliensis* (0.0723–0.0919) (Liu *et al*., 2024*a*, *b*). We therefore evaluate its genetic diversity as moderate to high when contrasted with prior research on other endangered plant species evaluated through ddRAD-seq. Meanwhile, all *C. yunnanensis* populations except PE had *H*_o_ values that were lower than *H*_e_ values, indicating a loss of genetic diversity during this species’ evolutionary process, and also align with the findings of research based on AFLP and SSR markers (Yang *et al*., 2016; Chen, 2020). The moderate to high genetic diversity observed in this species might be attributed to factors including its relatively broad distribution range. Additionally, being a relict plant species could play a role, with the surviving populations possibly representing just a small portion of what was once a more extensively distributed population. (Ren *et al*., 2024). Moreover, the low π value (0.0347) observed in the PE population indicates possible genetic relatedness between the two individuals within this population, which in turn leads to reduced nucleotide diversity. Although there are only four individuals in DB, the π value (0.0812) of this population is very high, suggesting that there may be more individuals distributed in the adjacent Vietnam region that we have not found. There is no clear correlation between population size and the contribution of genetic diversity, which may also be related to insufficient population surveys of this species.

### Genetic structure and genetic differentiation

By analysing the genetic structure, genetic differentiation and gene flow of threatened species, we can better understand the genetic background, and can therefore better assign genetic conservation units and propose scientific conservation strategies (Theissinger *et al*., 2023; Cai *et al*., 2024). The 12 populations of *C. yunnanensis* were classified into two optimal clusters through Admixture, PCA and DAPC, namely the eastern population (DS, DB, XJC, FD, XC, WMP and PE) and the western population (JD, HG, SD, LS and DX) with high differentiation. We attempted to select different *K* values to observe groupings within the different populations, but the results were difficult to analyse. The clustering results of *C. yunnanensis* also provide further evidence that the ‘Tanaka–Kaiyong Line’ (TKL) is a major phytogeographical boundary for driving population differentiation and phylogenetic geography of species in southwestern China, which corroborates results of studies into *Bombax ceiba* and *Sophora davidii* (Fan *et al*., 2013; Ju *et al*., 2018). As a relict plant species, the distribution of *C. yunnanensis* extends from the Himalayan region to the karst areas of Yunnan, Guizhou and Guangxi, and there has been significant genetic differentiation among populations. This result also provides new insights into the genetic isolation and population differentiation of the TKL in plant species with extremely small population groups.

The low genetic differentiation of *C. yunnanensis* populations indicates potentially high gene flow among various subpopulations, despite the fragmented distribution and small population, even with short distances flown by the effective pollinator (*Chrysomyia megacephala*) (Gao *et al*., 2012). This insect, which aims to steal nectar, stays on flowers for a long time and repeatedly visits the same flower. However, its effective pollination range usually does not exceed 2 km (Gao *et al*., 2012). According to our observations, *C. yunnanensis* trees can bloom and bear fruit after 8–10 years. The highest genetic differentiation values are between the DX and PE populations, which can be explained by the fact that the PE population, located in the Ailao Mountain area, is far from the DX population, which is located on the southern slope of the Himalayas. Of course, the small number of individuals in the PE population (*n* = 2 individuals) may also affect our analysis results (Supplementary Data Table S1). In contrast, the low genetic differentiation value and short geographical distance suggest that there may be relatively high gene flow between the XJC and FD populations. Of the existing populations, DS is located at the easternmost point of the karst area in Guizhou Province, and DX is located at the westernmost point of the southern Himalayan slope of Xizang (Fig. 1A). Interestingly, when we explored the population structure of *C. yunnanensis* under different *K* values, we found that the DS and DX populations maintain unique genetic backgrounds (Fig. S4). Therefore, we propose that particular attention should be paid to these two populations which are the most distant spatially when dividing genetic units.

In *C. yunnanensis*, geographical distance is significantly and positively correlated with genetic variation, a phenomenon that is often consistent with isolation by distance (Fig. 2D, E, F). That is, populations that are farther apart tend to have reduced gene flow due to geographical barriers, leading to increased genetic differentiation (Liu *et al*., 2024*a*, *b*). Conversely, the lack of a significant correlation between environmental factors and genetic variation implies that other non-environmental factors might be more influential in driving the observed genetic variation. The significant correlation between environmental factors and geographical distance may imply that there is a certain gradient of environmental factors across space, a variation that is related to the geographical separation between populations. As geographical locations vary, environmental conditions such as temperature and precipitation may undergo significant changes, which in turn can impact the distribution and genetic structure of organisms to a certain extent (Zhang *et al*., 2019).

### Demographic history

Understanding the population demographic history of threatened species can enhance their integrated conservation (Ma *et al*., 2022; Liu *et al*., 2024*a*, *b*). Without a reference genome, we thus chose the Stairway Plot 2 software with better testing performance (Cristofari *et al*., 2018; Liu and Fu, 2020; Sakaguchi *et al*., 2021; Wu *et al*., 2024). Although the study of the population demographic history of *C. yunnanensis* has not accurately inferred any bottleneck events in the history of this species, an effective population size value (*Ne*) between 10 and 20 ka was identified, consistent with the end of the Last Glacial Period (11.5 ka) and the Last Glacial Maximum (19–26.5 ka). For approximately 10 000 years thereafter, the *Ne* value remained at a high and stable level, which can explain the moderate to high genetic diversity we observed. In addition, Stairway Plot uses a one-dimension SFS of a single population, and the result could be affected largely by population structure, so we overlaid the data to present the total species results. A study of *Sophora davidii* (Vetchleaf Sophora) has also suggested that the last glaciation influenced the formation of different regional lineages in the eastern TKL (Fan *et al*., 2013), which can also be inferred in this study. Through extensive field investigations, we found that the current number of individuals in the wild of *C. yunnanensis* is fewer than 500, which differs significantly from the estimated effective population size (Fig. 3). Apart from the threat of inbreeding depression by low genetic differentiation, the main threats currently faced by *C. yunnanensis* are the destruction of habitats and individuals due to serious human interference, which are also the main reasons why the populations of *C. yunnanensis* are so small (Sun *et al*., 2019*b*).

### Outliers of natural selection

East Asia’s Tertiary relict plants have developed a unique genetic structure through long-term environmental changes and natural selection (Cao *et al*., 2016). To better respond to climatic fluctuations and habitat changes since the late Miocene, relict plants such as *Circaeaster agrestis* (star-leaf grass), *Pterocarya stenoptera* (Chinese wingnut) and *Davidia involucrata* (dove tree) all have selective sites related to environmental adaptability, providing important molecular evidence for elucidating the genetic variation and environmental adaptability of relict plants (Zhang *et al*., 2020; Wang *et al*., 2023; Ren *et al*., 2024). Common key factors determining the growth and distribution of species include temperature and precipitation. In the study of *C. yunnanensis*, we found that temperature had a more significant impact on its growth and distribution compared to precipitation, and the bioclimatic variables have a certain degree of non-independence. Outlier detection based on two different methods can effectively identify outliers in molecular data for this species. We found that most outliers in the *C. yunnanensis* data were associated with BIO19 (precipitation of coldest quarter), amounting to 1598 markers. The frequent occurrence of super-droughts in southwestern China in recent decades may reflect the shaping of this adaptive feature by natural selection (Wang *et al*., 2016).

### Genetic vulnerability under future climate scenarios

Against the backdrop of global climate change, rapid climate changes can sometimes exceed the adaptive capacity of plants, leading to a decrease in population numbers and even extinction (Capblancq *et al*., 2020). The population structure of species is correlated with the genetic offset among different populations. However, among the common analytical methods used, environmental factors are usually used to perform association analysis only with SNP loci (Sang *et al*., 2022; Zhu *et al*., 2024). Due to the distribution of *C. yunnanensis* in monsoon evergreen broad-leaved forests ranging from the Himalayas to the karst areas of Yunnan, Guizhou and Guangxi, which are relatively fragile habitats, corresponding strategies are needed based on genetic vulnerability analysis. Therefore, understanding how *C. yunnanensis* responds to future climate change is crucial for developing rational conservation strategies.

Previous studies have predicted the genetic mismatch of certain populations of threatened species in response to future environmental fluctuations by calculating the genetic offset of their populations, and have used this information to propose priority actions for their protection (Yang *et al*., 2022*b*; Shen *et al*., 2024). Prediction of genetic vulnerability in *Pterocarya macroptera* (Gansu wingnut) and *Cupressus gigantea* (Tsangpo cypress) populations suggests that marginal populations are more vulnerable and face a greater risk of extinction from future environmental fluctuations compared to central populations. (Yang *et al*., 2022*b*; Wang *et al*., 2023). In *C. yunnanensis*, the marginal populations (such as DS, DX) do not have significantly higher genetic offset than other populations, although the DX population exhibits high genetic offset values at all SNP loci. For populations to exist they need to be composed of a minimum population size in a protected habitat even when the genetic offset is low. They may also need more individuals for long-term successful persistence.

### Implications for conservation and management of *C. yunnanensis*

For the conservation and management of *C. yunnanensis*, we need to consider multiple factors such as habitat, population and genetic background. Fragmented habitats, coupled with severe human interference, have led to the endangered status of *C. yunnanensis*, with its small populations. From our field investigations, the main threats to *C. yunnanensis* include: (1) population isolation and significant habitat fragmentation due to the destruction of the original habitat; (2) small population sizes with a high proportion of ancient trees; and (3) the monsoon evergreen broad-leaved forest where *C. yunnanensis* is distributed is suitable for planting an economic crop, *Amomum tsaoko* (Tsaoko amomum fruit), which prevents natural regeneration. In response to these threats, our proposed conservation measures are: (1) to carry out *in situ* conservation for populations and individuals outside the natural reserve as soon as possible, such as populations DB, DS, DX and PE; and (2) to prevent the planting of economic crops in the original *C. yunnanensis* habitat, such as populations XC, XJC and LS; (3) targeted conservation actions such as translocation, reintroduction or population enhancement are needed for most populations. Furthermore, given the genetic backgrounds of different populations, the germplasm resources of different populations need to be collected and conserved, especially for isolated populations and populations with unique genetic backgrounds, such as DS and DX. Populations with small numbers of individuals, including DB, XC and PE, face a high risk of extinction, and for these populations, we need to conduct deeper genetic research such as genetic load analysis based on whole genome resequencing data to detect mutation sites and accumulation of harmful mutations in different populations and propose artificial outcrossing strategies to reduce the loss of genetic diversity caused by inbreeding or selfing, genetic decline in offspring and decreased survival ability (Frankham *et al*., 2017; Yang *et al*., 2018; Chen, 2020; Cai *et al*., 2024). To avoid potential outbreeding depression, the germplasm sources of different populations should be mixed with caution taking into account environmental differences among populations (Frankham *et al*., 2011, 2017; Yang *et al*., 2016; Chen, 2020). Based on genetic background, a reasonable *ex situ* conservation population should be constructed in a suitable location, which is also an important measure to maintain the genetic diversity (Mounce *et al*., 2017; Xiong *et al*., 2024). Through genetic vulnerability analysis, we found that the *C. yunnanensis* populations are not significant to environmental change in the future. In addition, due to various reasons, insufficient sample sizes and incomplete geographical coverage in many of these populations may limit the ability to predict future climate change scenarios.

## Supplementary Material

mcaf270_Supplementary_Data

## SUPPLEMENTARY DATA

Supplementary data are available at *Annals of Botany* onlineand consist of the following. Table S1: statistical analysis of sequencing data of *Craigia yunnanensis* by ddRAD. Table S2: CV error values corresponding to different *K* values. Table S3: redundancy analysis for GEA (RDA-GEA). Table S4: latent factor mixed modelling for GEA (LFMM-GEA). Figure S1: the weight of 19 environmental factors. Figure S2: Pearson’s correlation analysis of the 19 bioclimatic variables. Figure S3: CV error values corresponding to different *K* values. Figure S4: Population structure of *Craigia yunnanensis* from 12 subpopulations inferred by Stacks data sets. Admixture results with (A) *K* = 3, (B) *K* = 4, (C) *K* = 5, (D) *K* = 6 and (E) *K* = 7. Figure S5: correlation analysis of the offset values of the eastern and western lineages under different scenarios.
