# Supplementary material for: Conservation genetics and genetic vulnerability of Craigia yunnanensis (Malvaceae), a relict plant species with extremely small populations from Southwest China
Source: Ann Bot. 2025 Oct 30;138(1):88–100. doi: 10.1093/aob/mcaf270 (PMC13409162; doi:10.1093/aob/mcaf270)
Supplement: mcaf270_Supplementary_Data [file mcaf270_supplementary_data.zip › Supplementary Data Fig. S1-S5.docx]

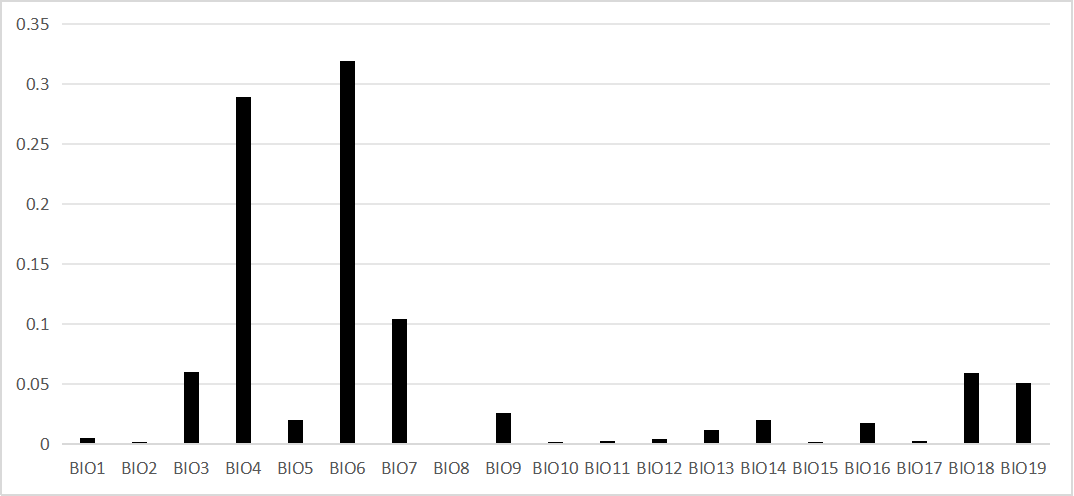


**Fig. S1** The weight of 19 environmental factors

BIO1: Annual mean temperature (℃); BIO2: Mean diurnal temperature range (℃); BIO3: Isothermality (BIO2/BIO7) (×100); BIO4: Temperature seasonality (standard  deviation ×100); BIO5: Maximum temperature of warmest month (℃); BIO6: Minimum temperature of coldest month (℃); BIO7: Temperature annual range (℃); BIO8: Mean temperature of wettest quarter (℃); BIO9: Mean temperature of driest quarter (℃); BIO10: Mean temperature of warmest quarter (℃); BIO11: Mean temperature of coldest quarter (℃); BIO12: Annual precipitation (mm); BIO13: Precipitation of wettest month (mm); BIO14: Precipitation of driest month (mm); BIO15: Precipitation seasonality (mm) (Coefficient of Variation); BIO16: Precipitation of wettest quarter (mm); BIO17: Precipitation of driest quarter (mm); BIO18: Precipitation of warmest quarter (mm); BIO19: Precipitation of coldest quarter (mm).


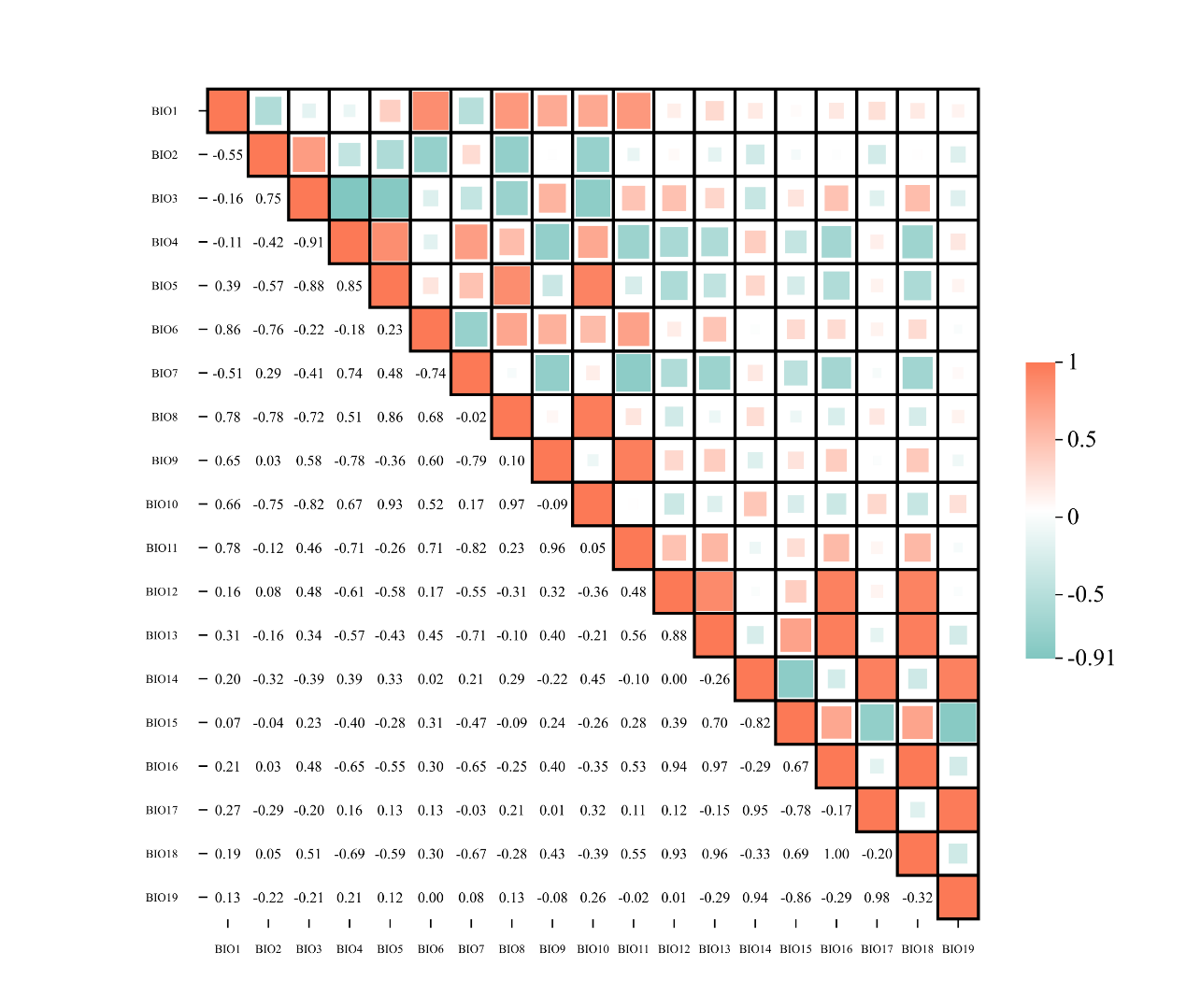


**Fig. S2** Pearson’s correlation analysis of the 19 bioclimatic variables


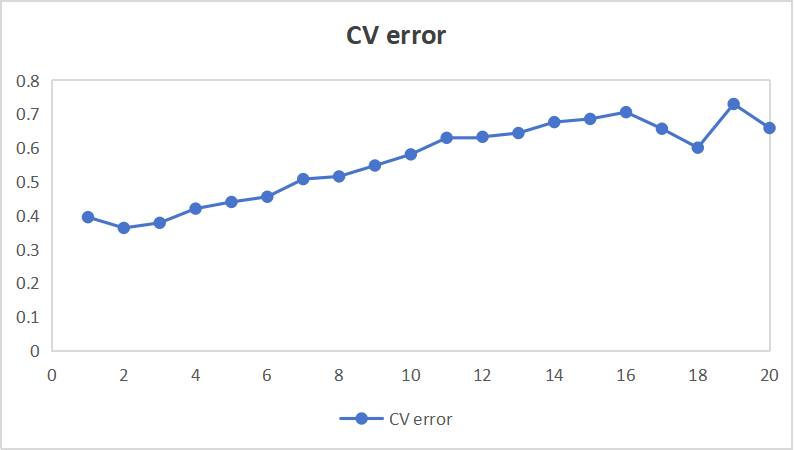


**Fig. S3** CV error values corresponding to different K values


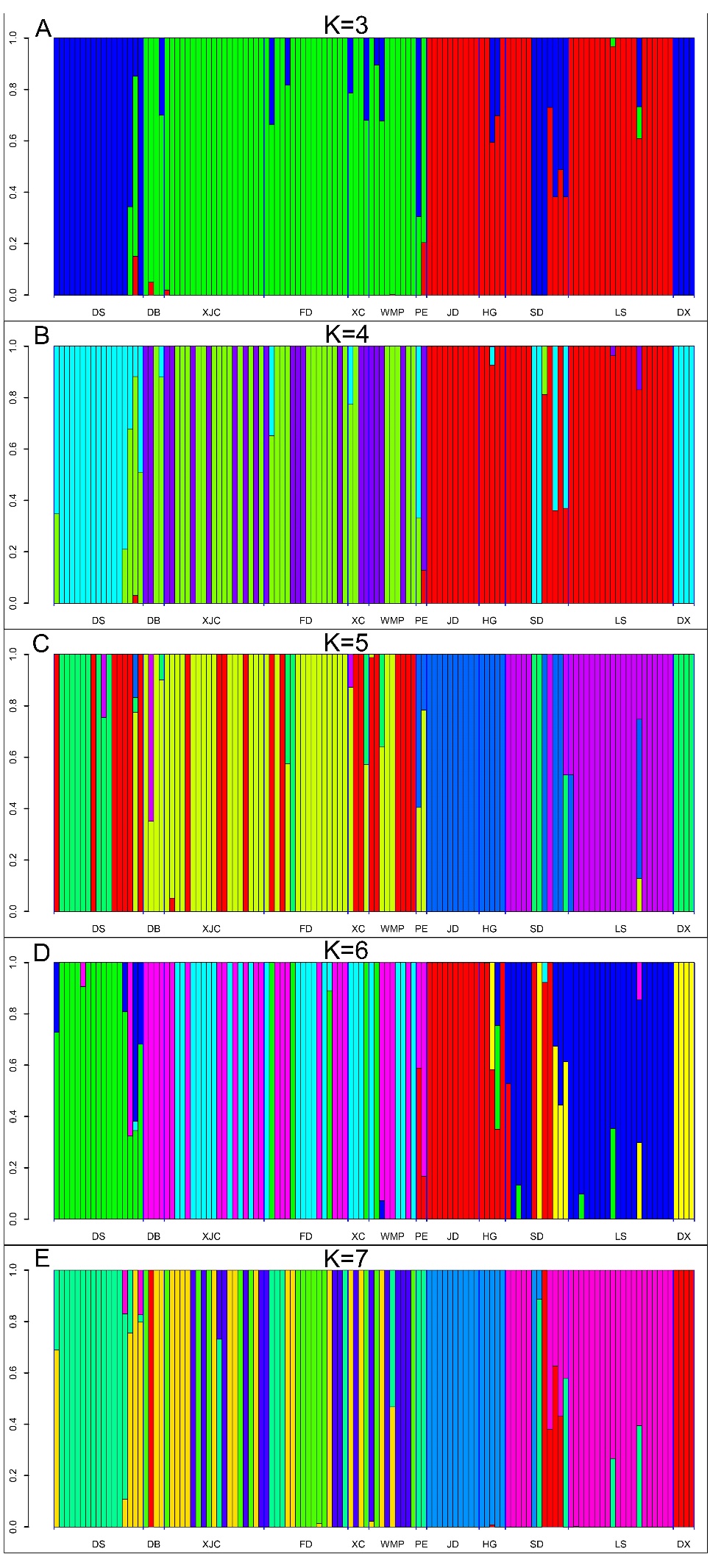


**Fig. S4** Population structure of *Craigia yunnanensis* from 12 subpopulations inferred by Stacks data sets. Admixture results with (A) K = 3, (B) K = 4, (C) K=5, (D) K=6 and (E) K=7


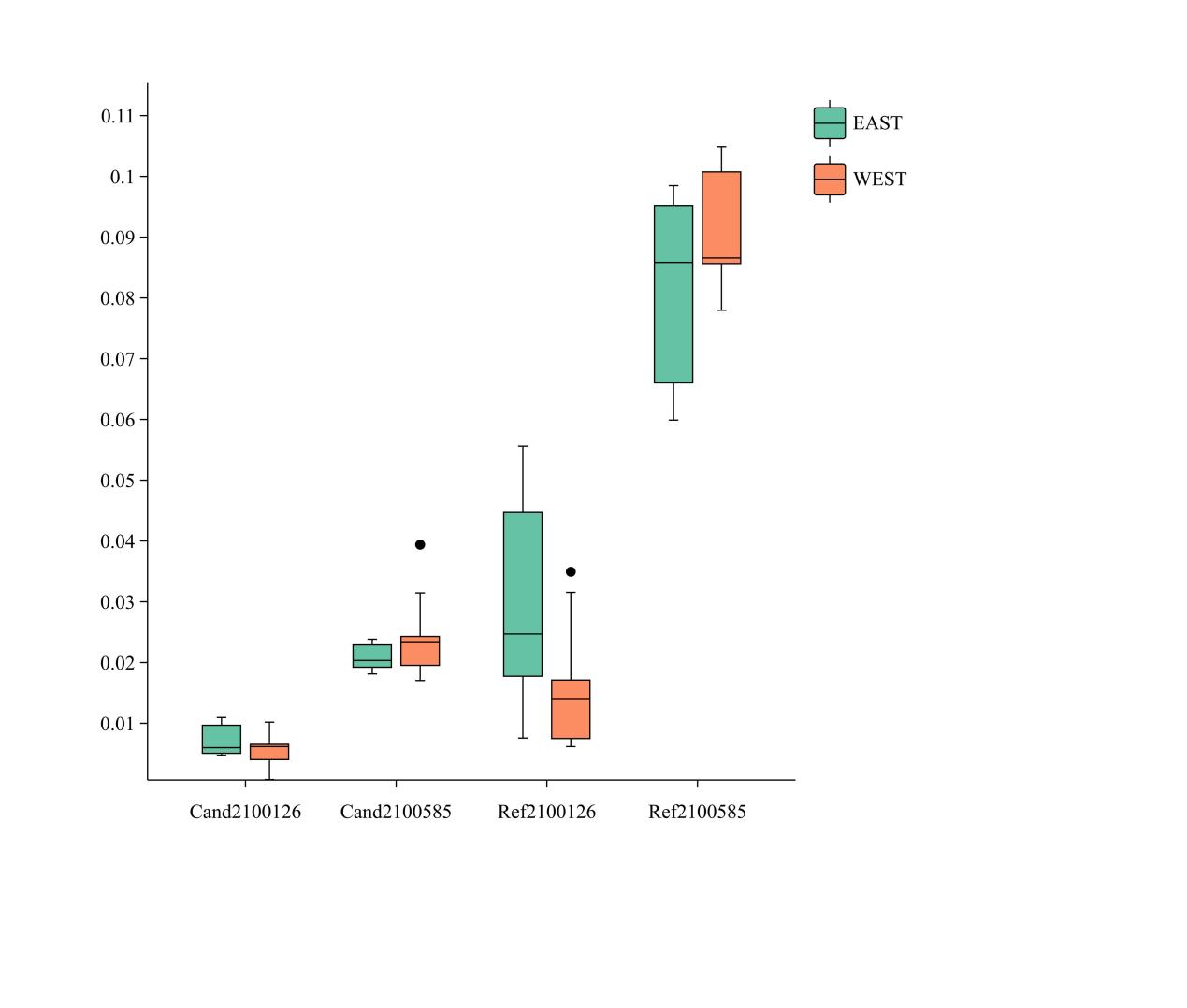


**Fig. S5** Correlation analysis of the offset values of the eastern and western lineages under different scenarios.
